# Performance of lupus classification criteria in monogenic lupus: a national multicentre cohort study

**DOI:** 10.1093/rheumatology/keag532

**Published:** 2026-10-05

**Authors:** Nergis Akay, Sezgin Sahin, Sila Atamyildiz Ucar, Aybuke Alisan, Eray Tunce, Eda Kayhan, Yasemin Ugur Es, Aysenur Alkaya, Busra Baser Taskin, Dilara Unal, Lutfiye Koru, Hatice Kubra Zora, Aslihan Uzun Bektas, Melisa Kacmaz, Halil Kazanasmaz, Sengul Caglayan, Seher Sener, Busra Acun, Gulcan Ozomay Baykal, Yasemin Yigit Demir, Suleyman Ekrem Albayrak, Burcu Bozkaya, Orhan Nurlu, Mehmet Yildiz, Amra Adrovic, Kenan Barut, Sevcan A Bakkaloglu, Metin Kaya Gurgoze, Ayse Balat, Fatih Haslak, Balahan Bora, Sara Sebnem Kilic, Mukaddes Kalyoncu, Oya Koker, Semanur Elmas, Nuray Aktay Ayaz, Seza Ozen, Banu Celikel Acar, Sumeyra Ozdemir Cicek, Sibel Balci, Aysenur Pac Kisaarslan, Rabia Miray Kisla Ekinci, Betul Sozeri, Ozgur Kasapcopur

**Affiliations:** Department of Paediatric Rheumatology, Istanbul University-Cerrahpasa, Cerrahpasa Faculty of Medicine, Istanbul, Türkiye; Department of Paediatric Rheumatology, Istanbul University-Cerrahpasa, Cerrahpasa Faculty of Medicine, Istanbul, Türkiye; Department of Paediatric Rheumatology, Umraniye Training and Research Hospital, University of Health Sciences, Istanbul, Türkiye; Department of Paediatric Rheumatology, Cukurova University Faculty of Medicine, Adana, Türkiye; Department of Paediatric Rheumatology, Umraniye Training and Research Hospital, University of Health Sciences, Istanbul, Türkiye; Department of Paediatric Rheumatology, Erciyes University Faculty of Medicine, Kayseri, Türkiye; Department of Paediatric Rheumatology, University of Health Sciences, Ankara Bilkent City Hospital, Ankara, Türkiye; Department of Paediatric Rheumatology, University of Health Sciences, Etlik City Hospital, Ankara, Türkiye; Department of Paediatric Rheumatology, Istanbul University Faculty of Medicine, Istanbul, Türkiye; Department of Paediatric Rheumatology, Hacettepe University Faculty of Medicine, Ankara, Türkiye; Department of Paediatric Rheumatology, Istanbul Medeniyet University Faculty of Medicine, Istanbul, Türkiye; Department of Paediatric Immunology and Rheumatology, Uludag University Faculty of Medicine, Bursa, Türkiye; Department of Paediatric Rheumatology, Dokuz Eylul University Faculty of Medicine, Izmir, Türkiye; Department of Paediatric Rheumatology, Diyarbakır Children’s Hospital, Diyarbakır, Türkiye; Department of Paediatric Rheumatology, Karadeniz Technical University Faculty of Medicine, Trabzon, Türkiye; Department of Paediatric Rheumatology, Antalya Training and Research Hospital, Antalya, Türkiye; Department of Paediatric Rheumatology, Adana City Training and Research Hospital, Adana, Türkiye; Department of Paediatric Rheumatology, Gazi University Faculty of Medicine, Ankara, Türkiye; Department of Paediatric Rheumatology, Mardin Training and Research Hospital, Mardin, Türkiye; Department of Paediatric Rheumatology, Gazi Yasargil Training and Research Hospital, Diyarbakır, Türkiye; Department of Paediatric Rheumatology, Fırat University Faculty of Medicine, Elazığ, Türkiye; Department of Paediatric Rheumatology, Samsun Training and Research Hospital, Samsun, Türkiye; Department of Paediatric Rheumatology, Gaziantep University Faculty of Medicine, Gaziantep, Türkiye; Department of Paediatric Rheumatology, Istanbul University-Cerrahpasa, Cerrahpasa Faculty of Medicine, Istanbul, Türkiye; Department of Paediatric Rheumatology, Istanbul University-Cerrahpasa, Cerrahpasa Faculty of Medicine, Istanbul, Türkiye; Department of Paediatric Rheumatology, Istanbul University-Cerrahpasa, Cerrahpasa Faculty of Medicine, Istanbul, Türkiye; Department of Paediatric Rheumatology, Gazi University Faculty of Medicine, Ankara, Türkiye; Department of Paediatric Rheumatology, Fırat University Faculty of Medicine, Elazığ, Türkiye; Department of Paediatric Rheumatology, Gaziantep University Faculty of Medicine, Gaziantep, Türkiye; Department of Paediatric Rheumatology, Istanbul Medeniyet University Faculty of Medicine, Istanbul, Türkiye; Department of Paediatric Rheumatology, Dokuz Eylul University Faculty of Medicine, Izmir, Türkiye; Department of Paediatric Immunology and Rheumatology, Uludag University Faculty of Medicine, Bursa, Türkiye; Department of Paediatric Rheumatology, Karadeniz Technical University Faculty of Medicine, Trabzon, Türkiye; Department of Paediatric Rheumatology, Marmara University Faculty of Medicine, Istanbul, Türkiye; Department of Paediatric Rheumatology, Samsun Training and Research Hospital, Samsun, Türkiye; Department of Paediatric Rheumatology, Istanbul University Faculty of Medicine, Istanbul, Türkiye; Department of Paediatric Rheumatology, Hacettepe University Faculty of Medicine, Ankara, Türkiye; Department of Paediatric Rheumatology, University of Health Sciences, Ankara Bilkent City Hospital, Ankara, Türkiye; Department of Paediatric Rheumatology, Erciyes University Faculty of Medicine, Kayseri, Türkiye; Department of Paediatric Rheumatology, Dicle University Faculty of Medicine, Diyarbakır, Türkiye; Department of Paediatric Rheumatology, Erciyes University Faculty of Medicine, Kayseri, Türkiye; Department of Paediatric Rheumatology, Cukurova University Faculty of Medicine, Adana, Türkiye; Department of Paediatric Rheumatology, Umraniye Training and Research Hospital, University of Health Sciences, Istanbul, Türkiye; Department of Paediatric Rheumatology, Istanbul University-Cerrahpasa, Cerrahpasa Faculty of Medicine, Istanbul, Türkiye

**Keywords:** monogenic lupus, early-onset autoimmunity, complement deficiency, classification

## Abstract

**Objectives:**

With advances in genetic testing, monogenic lupus is increasingly recognized, yet disease-specific classification criteria are lacking. We compared all three classification criteria sets in the same monogenic lupus cohort and, for the first time, evaluated their performance across distinct genetic pathways.

**Methods:**

This national multicentre study included 88 genetically confirmed monogenic lupus patients from 22 paediatric rheumatology centres across Türkiye. Fulfilment of the AmericanCollege of Rheumatology (ACR)-1997, Systemic Lupus International Collaborating Clinics (SLICC)-2012 and European League Against Rheumatism (EULAR)/ACR-2019 criteria and pathway-based differences in classification performance were assessed.

**Results:**

Overall, 74% (*n* = 65) fulfilled ACR-1997, 70.5% (*n* = 62) SLICC-2012 and EULAR/ACR-2019 criteria, and 62.5% (*n* = 55) met all three, whereas 17% (*n* = 15) met none. Agreement was substantial (κ = 0.63–0.73), with no significant differences between criteria (*P > *0.05). Classification performance differed significantly across genetic pathways (*P* < 0.05), with the highest fulfilment performances in complement deficiencies and the lowest in nucleic acid metabolism and type I interferon pathway defects. Most patients who did not fulfil any classification criteria had nucleic acid metabolism and clearance defects (13/15, 86.7%). Domain-level analyses showed that classification success was mainly driven by mucocutaneous, immunologic and renal domains. Complement and immune-regulation defects showed a more classical lupus phenotype, with most fulfilling all three criteria (78.6%, 22/28 and 77.8%, 14/18), whereas the proportion was lower in type I interferon pathway and nucleic acid metabolism and clearance defects (33.3%, 2/6 and 47.2%, 17/36). No classification criteria were superior within genetic pathways.

**Conclusion:**

One-third of genetically confirmed monogenic lupus patients remained unclassified, particularly those with interferon-related and nucleic acid metabolism defects, highlighting pathway-dependent limitations of current classification criteria.

## Graphical abstract

**Graphical Abstract:**
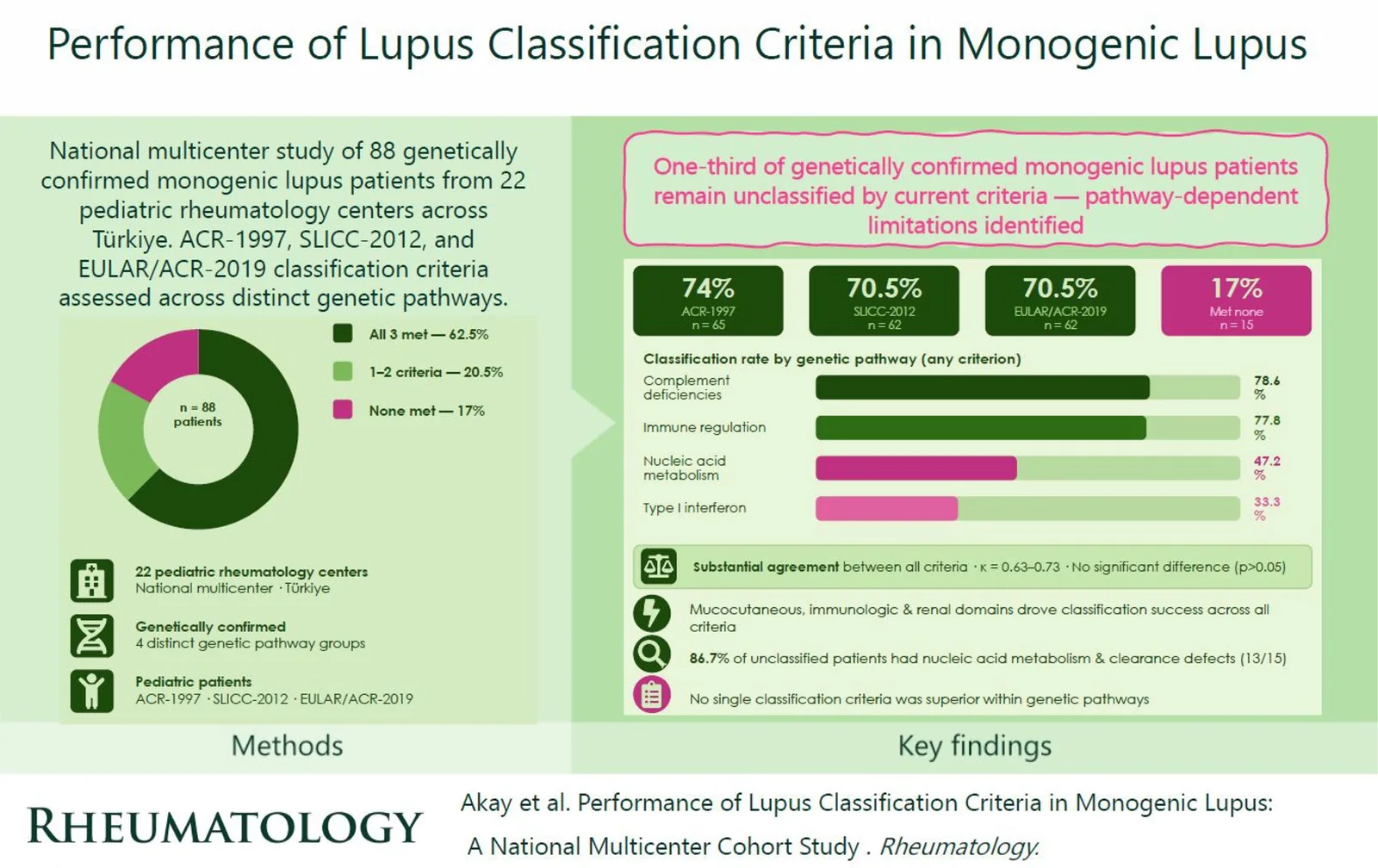

*Rheumatology* key messages

- This study compares three SLE classification criteria across genetically defined monogenic lupus pathways.
- One-third of paediatric monogenic lupus patients were not classified by any SLE criteria.
- Genetics-informed classification may better capture the clinical and biological diversity of monogenic lupus.

## Introduction

Systemic lupus erythematosus (SLE) is a multisystem autoimmune connective tissue disease driven by genetic susceptibility in combination with environmental and epigenetic factors and predominantly affects females across both paediatric and adult populations [1, 2]. Monogenic lupus represents a rare but distinct subset of SLE that typically presents in early childhood, often around 5 years of age, and accounts for ∼1–2% of all lupus cases and up to 7% of childhood-onset lupus [3–5]. It arises from pathogenic variants in single genes that disrupt specific immune pathways, frequently resulting in a more severe and atypical clinical phenotype. Advances in high-throughput sequencing have led to the identification of >30 genes associated with monogenic lupus, many of which converge on innate and adaptive immune mechanisms, including complement regulation, nucleic acid sensing, type I interferon (IFN-I) signalling, and T- and B-cell activation [3–7].

Clinically, very early disease onset, consanguinity, male sex, strong family history, severe or atypical organ involvement and poor response to conventional immunosuppressive therapy should raise suspicion for monogenic lupus [6]. However, marked clinical heterogeneity and overlap with both classical SLE and monogenic interferonopathies, together with the absence of validated classification criteria, pose significant diagnostic challenges. Although the EULAR/ACR-2019 and SLICC-2012 classification criteria have previously been evaluated in monogenic lupus cohorts, the comparative performance of all three major classification criteria sets (ACR-1997, SLICC-2012 and EULAR/ACR-2019) have not been assessed within the same cohort or across genetically defined pathway subgroups [8, 9].

To address this gap, we performed a nationwide multicentre study evaluating the performance of the ACR-1997, SLICC-2012 and EULAR/ACR-2019 classification criteria in a genetically confirmed paediatric monogenic lupus cohort, with a particular focus on pathway-dependent differences in classification criteria sets performance, representing the first pathway-oriented comparative analysis in this field.

## Material and methods

### Study design

This multicentre observational study was conducted across 22 paediatric rheumatology centres in Türkiye. Clinical and demographic data were retrospectively extracted from patient medical records and institutional electronic hospital systems at each participating site. All data were recorded in a centralized, secure electronic database specifically developed for this study using the REDCap platform, with institutional support from Cerrahpaşa Faculty of Medicine. To ensure consistency and data quality, standardized electronic case report forms were used across all centres. Institutional approval was obtained from all participating centres confirming that patient data would be fully anonymized prior to submission to the ethics committee, and the study protocol was subsequently reviewed and approved by Istanbul University – Cerrahpasa Clinical Research Ethics Committee (approval date: 28 October 2025, approval number: 1463078) and conducted in accordance with the Helsinki Declaration.

### Study population

Patients were included based on the following criteria: diagnosis before 18 years of age; presence of any clinical feature compatible with lupus or lupus-like disease; and identification of a pathogenic, likely pathogenic or Variant of Uncertain Significance (VUS) variant in a gene associated with monogenic lupus by whole-exome sequencing (WES) or targeted exome sequencing (TES), with no alternative diagnosis explaining the clinical findings. Patients without regular follow-up or with >50% missing clinical data were excluded. Despite carrying variants known to cause monogenic lupus-associated phenotypes, patients who had never been referred to paediatric rheumatology due to the absence of any rheumatological/lupus manifestation and were followed solely by immunology and/or neurology clinics were excluded.

### Data collection

Fulfilment of individual classification items and other lupus-like manifestations were assessed cumulatively over the entire disease course; a clinical feature or laboratory finding was considered positive if documented at any point during follow-up.

Clinical and serological features present at any time of disease course were retrospectively reviewed in this multicentre national cohort. Manifestations corresponding to conventional SLE classification domains including mucocutaneous, hematologic, renal, neuropsychiatric, arthritis and serositis involvement were recorded. Serological parameters (ANA, anti-dsDNA, anti-Sm, direct Coombs test, complement levels [C3, C4] and antiphospholipid antibodies) were extracted from medical records to evaluate fulfilment and performance of established SLE classification criteria sets including ACR-1997, SLICC-2012 and EULAR/ACR-2019 [10–12].

All genetic analyses were performed using WES or TES. Sanger sequencing was applied for patients with a previously identified familial pathogenic variant. Pathogenic, likely pathogenic and VUS variants were identified and recorded. Patients were subsequently stratified according to pathway-defined subgroups based on the primary molecular mechanisms reported in the literature as follows: complement deficiencies, defects in nucleic acid metabolism and clearance, type I interferon signalling abnormalities, and disorders of immune regulation and lymphocyte. The performance of established SLE classification criteria sets was evaluated both in the all-monogenic lupus cohort and across pathway-defined subgroups [1, 7, 13–15].

### Statistical analysis

All statistical analyses were performed using IBM SPSS Statistics (version 26). Continuous variables were assessed for normality using the Kolmogorov–Smirnov test and visual inspection of histograms. Normally distributed variables were presented as mean ± standard deviation, whereas non-normally distributed variables were summarized as median with interquartile ranges (Q1–Q3). Categorical variables were expressed as frequencies and percentages. Agreement between classification criteria was evaluated using Cohen’s kappa coefficient. Paired proportions were compared using McNemar’s test and pairwise comparisons were adjusted for multiple testing using Bonferroni correction. A kappa value of 0.61–0.80 was considered to indicate substantial agreement. Comparisons of continuous variables among genetic pathway groups were performed using the Kruskal–Wallis test. Associations between genetic pathway categories and categorical variables were analysed using Pearson’s χ^2^ or Monte Carlo exact tests, as appropriate. A two-sided *P*-value <0.05 was considered statistically significant. All graphs were generated using GraphPad Prism, version 10.6.1 (799) (GraphPad Software, Boston, MA, USA).

## Results

### Baseline characteristics of the monogenic lupus cohort

Pathogenic, likely pathogenic and VUS variants identified across the 88-patient cohort were classified into four major functional genetic pathways based on their underlying molecular mechanisms. Complement pathway deficiencies, including variants in *C1QA*, *C1QB, C1QC*, *C1R*, *C1S*, *C2*, *C4A*, *CFB* and *CR2* were identified in 28 patients (31.8%). Variants affecting nucleic acid metabolism and clearance (*DNASE1L3*, *DNASE1*, *TREX1* and *SAMHD1*) were detected in 36 patients (40.9%). Type I interferon signalling and regulatory pathway variants (*DDX58*, *IFIH1*, *ADAR*, *IRF5* and *IRAK1*) were present in six patients (6.8%). Finally, variants associated with immune regulation and lymphocyte signalling [*ACP5*, *PRKCD*, *PRKCQ*, *STAT3* (gain-of-function), *SOCS1*, *SHOC2*, *ZAP70*, *RAG1*, *HDAC7*, *FOXP3* and *RELA*] were identified in 18 patients (20.5%). Patients were subsequently stratified according to these pathway-defined subgroups for downstream analyses.

### Performance of classification criteria sets

Among 88 patients, 65 (74.0%) fulfilled the ACR-1997 criteria, while 62 (70.5%) met both the SLICC-2012 and EULAR/ACR-2019 criteria. Overall, 55 patients (62.5%) satisfied all three classification systems, whereas 15 (17.0%) were not captured by any criteria (Fig. 1A). Despite extensive multisystem involvement, 14 of 63 patients (22.2%) with three or more affected clinical domains still failed to meet EULAR/ACR-2019 criteria (Fig. 1B). Classification performance differed significantly across genetic pathway subgroups (*P = *0.004): complement pathway defects (*n* = 28) showed the highest rate (89.3%), followed by immune dysregulation (*n* = 18, 83.3%) and interferon signalling defects (*n* = 6, 66.7%), whereas nucleic acid metabolism defects (*n* = 36) demonstrated the lowest performance (50.0%), accounting for 69.2% of all unclassified patients (Fig. 1C).

**Figure 1:**
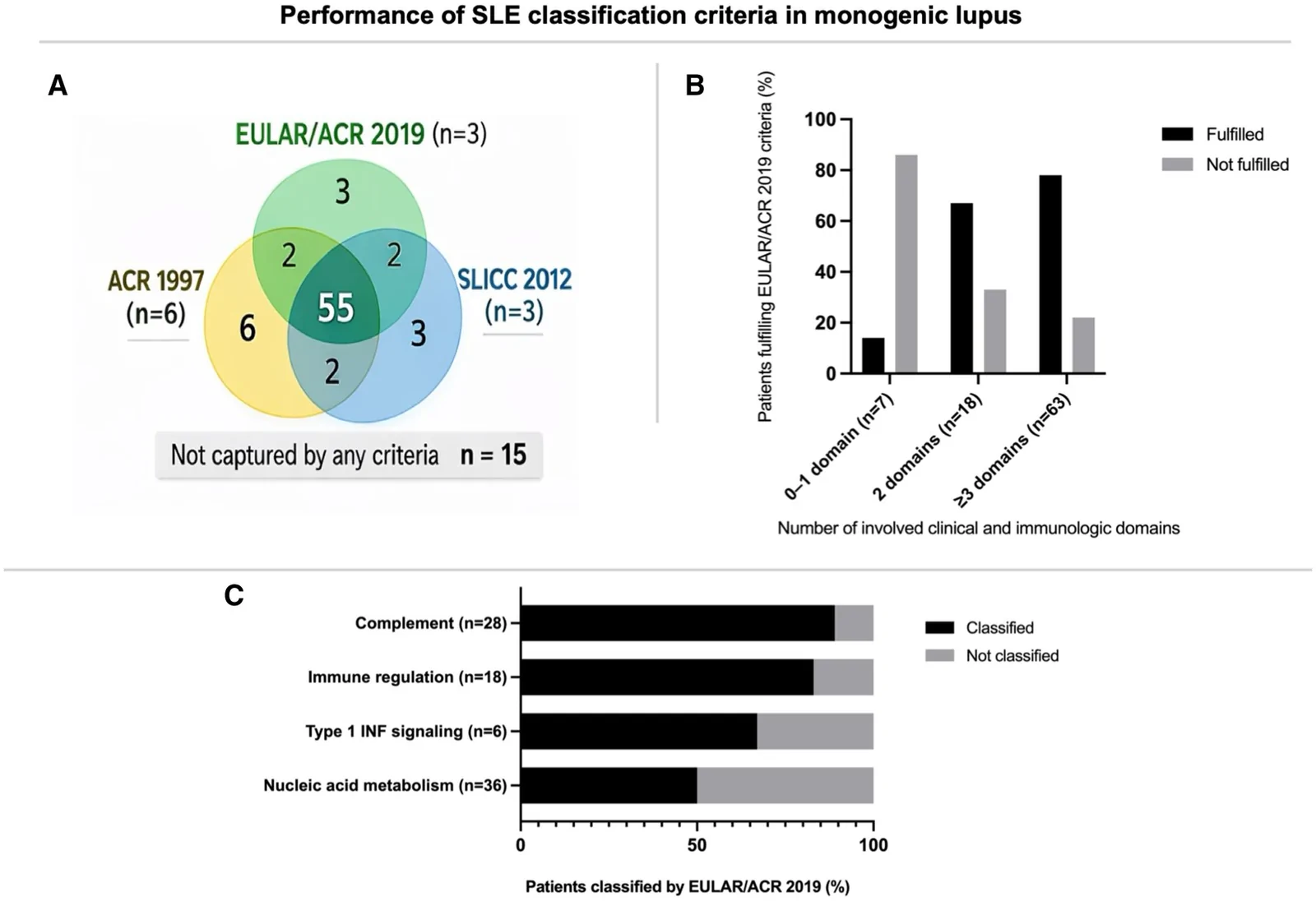
Three-panel figure illustrating the performance of SLE classification criteria in 88 patients with genetically confirmed monogenic lupus. Panel A shows a three-circle Venn diagram comparing the ACR-1997, SLICC-2012, and EULAR/ACR-2019 criteria. Fifty-five patients fulfilled all three sets, while 15 fulfilled none. Panel B shows a grouped bar chart demonstrating that fulfilment of the EULAR/ACR-2019 criteria increases with the number of involved clinical and immunological domains. Clinical system involvement was defined as the presence of at least one feature within the following domains: constitutional, mucocutaneous, musculoskeletal (arthritis), serositis, hematologic, renal or neurologic. Immunologic system involvement was defined as fulfilment of at least one immunologic domain, including antiphospholipid antibodies, complement or highly specific antibodies (anti-dsDNA or anti-Sm). Classification performances increased with greater multisystem involvement; however, a subset of patients with extensive disease remained unclassified. Panel C shows horizontal stacked bar charts comparing the fulfilment of the EULAR/ACR-2019 classification criteria across four genetic pathways. Classification rates were highest in complement pathway defects and lowest in nucleic acid metabolism disorders

Concordance between the SLICC-2012 and ACR-1997 criteria was observed in 75 of 88 patients (85.2%; κ = 0.63, *P* < 0.001), while agreement between EULAR/ACR-2019 and ACR-1997 criteria was similarly high (85.2%; κ = 0.63, *P* < 0.001). The highest concordance was found between the SLICC-2012 and EULAR/ACR-2019 criteria, with 78 of 88 patients (88.6%) classified consistently (κ = 0.73, *P* < 0.001). McNemar’s test demonstrated no significant differences in overall classification performance between the three classification criteria sets (all *P > *0.05).

### Differential domain capture by SLE classification criteria sets

Conventional SLE classification criteria demonstrated substantial variability in their ability to capture the clinical and immunological spectrum of monogenic lupus. Distinct domain distributions among uniquely classified and unclassified patients suggest that differences in domain weighting contribute significantly to classification discordance.

### ACR-1997

ACR-1997 classification was primarily driven by mucocutaneous and immunological features. Among patients fulfilling the criteria, malar rash (78.5% *vs* 17.4%), photosensitivity (70.8% *vs* 4.3%), oral ulcers (55.4% *vs* 8.7%) and discoid rash (26.6% *vs* 0%) were substantially more frequent compared with non-fulfilling patients (all *P* < 0.05). Renal involvement was also more prevalent in classified patients (43.1% *vs* 13.0%, *P = *0.010). The strongest associations were observed for immunological criteria (69.2% *vs* 13.0%, *P* < 0.001) and ANA positivity (90.8% *vs* 30.4%, *P* < 0.001), underscoring the immunological dominance of ACR-1997 fulfilment in this cohort (Table 1).

**Table 1:** Clinical and immunologic determinants of ACR-1997 classification fulfilment in genetically confirmed monogenic lupus.

ACR-1997	Fulfilled (*n* = 65, 74%)	Not fulfilled (*n* = 23, 26%)	*P* value
Malar rash	51 (78.5)	4 (17.4)	**<0.001**
Discoid rash	17 (26.6)	0 (0)	**0.004**
Photosensitivity	46 (70.8)	1 (4.3)	**<0.001**
Oral ulcers	36 (55.4)	2 (8.7)	**<0.001**
Arthritis	22 (33.8)	11 (47.8)	0.234
Serositis	12 (18.5)	3 (13)	0.750
Renal involvement	28 (43.1)	3 (13)	**0.010**
Neurologic involvement	13 (20)	2 (8.7)	0.336
Hematologic involvement	32 (49.2)	7 (30.4)	0.119
Immunologic involvement	45 (69.2)	3 (13.0)	**<0.001**
ANA positivity	59 (90.8)	7 (30.4)	**<0.001**

Data are presented as *n* (%). Comparisons between groups were performed using χ^2^ or Fisher’s exact test as appropriate. ANA positivity was evaluated as part of the immunologic criteria within the ACR-1997 classification system. Bolded *P* values <0.05 were considered statistically significant. ACR: American College of Rheumatology; ANA: antinuclear antibody.

### SLICC-2012

SLICC-2012 classification was driven by mucocutaneous, renal, haematologic and immunological features. Acute/subacute cutaneous lupus (72.6% *vs* 34.6%), chronic cutaneous lupus (35.5% *vs* 7.7%), oral/nasal ulcers (51.6% *vs* 19.2%) and non-scarring alopecia (33.9% *vs* 3.8%) were all significantly more frequent in classified patients (all *P* < 0.05). Renal involvement (45.2% *vs* 11.5%, *P = *0.003) and Class III–IV lupus nephritis (*P = *0.025) were markedly more prevalent among fulfilling patients. Among haematologic features, haemolytic anaemia (38.7% *vs* 11.5%, *P = *0.012) and leukopenia/lymphopenia (40.3% *vs* 15.4%, *P = *0.023) were significantly associated with SLICC-2012 fulfilment, whereas thrombocytopenia was not (*P = *0.287). Immunologically, ANA positivity (93.5% *vs* 30.8%), anti-dsDNA positivity (53.2% *vs* 0%), low complement levels (72.6% *vs* 42.3%) and direct Coombs positivity (58.5% *vs* 4.5%) were all significantly more frequent in classified patients (all *P* < 0.05). The coexistence of ANA positivity and lupus nephritis was observed exclusively among patients fulfilling SLICC-2012 criteria (32.3% *vs* 0%, *P < *0.001) (Table 2).

**Table 2:** Clinical and immunologic determinants of SLICC-2012 classification fulfilment in genetically confirmed monogenic lupus.

SLICC-2012	Fulfilled (*n* = 62, 70.5%)	Not fulfilled (*n* = 26, 29.6%)	*P* value
Acute/subacute cutaneous	45 (72.6)	9 (34.6)	**0.001**
Chronic cutaneous	22 (35.5)	2 (7.7)	**0.008**
Oral/nasal ulcers	32 (51.6)	5 (19.2)	**0.005**
Alopecia	21 (33.9)	1 (3.8)	**0.003**
Arthritis	22 (35.5)	11 (42.3)	0.546
Serositis	10 (16.1)	3 (11.5)	0.747
Renal involvement	28 (45.2)	3 (11.5)	**0.003**
Neurologic involvement	17 (27.4)	4 (15.4)	0.227
Haemolytic anaemia	24 (38.7)	3 (11.5)	**0.012**
Leukopenia/lymphopenia	25 (40.3)	4 (15.4)	**0.023**
Thrombocytopenia	16 (25.8)	8 (30.8)	0.287
ANA positivity	58 (93.5)	8 (30.8)	**<0.001**
Anti-dsDNA positivity	33 (53.2)	0 (0)	**<0.001**
Anti-Sm positivity	20 (32.2)	3 (11.5)	**0.044**
Antiphospholipid antibody positivity	13 (21)	0 (0)	**0.005**
Low complement	45 (72.6)	11 (42.3)	**0.007**
Direct Coombs positivity	31 (58.5)	1 (4.5)	**<0.001**
ANA + LN	20 (32.3)	0 (0)	**<0.001**

Data are presented as *n* (%). Comparisons between groups were performed using χ^2^ or Fisher’s exact test as appropriate. ANA positivity was assessed independently and does not constitute an entry criterion in the SLICC 2012 classification system. Bolded *P* values <0.05 were considered statistically significant. SLICC: Systemic Lupus International Collaborating Clinics; ANA: antinuclear antibody; Anti-dsDNA: anti–double-stranded DNA antibody; Anti-Sm: anti-Smith antibody; LN: lupus nephritis.

### EULAR/ACR-2019

Among clinical domains, mucocutaneous (77.4% *vs* 50.0%, *P = *0.011) and immunologic involvement (91.9% *vs* 57.7%, *P < *0.001) were significantly associated with EULAR/ACR-2019 fulfilment. All anti-dsDNA-positive patients met the criteria, and none of the ANA-negative patients fulfilled classification. Low complement levels were more frequent among classified patients (69.4% *vs* 50.0%) but did not reach significance (*P = *0.085) (Table 3). In the full cohort, mucocutaneous, immunologic, renal and constitutional domains significantly contributed to scores ≥10, whereas in ANA-positive patients, classification was predominantly driven by immunologic and renal domains (Fig. 2A and B).

**Figure 2:**
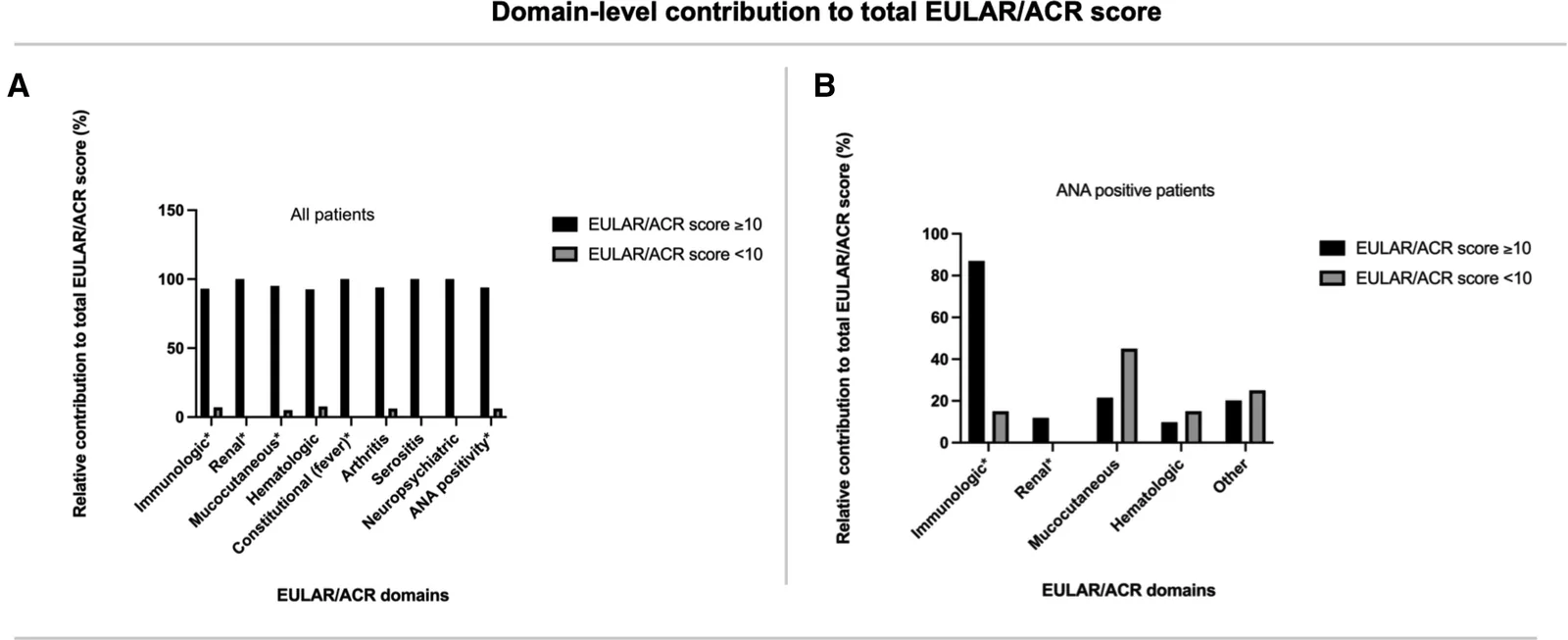
Domain-level contribution to total EULAR/ACR score according to classification threshold. (**A**) In the overall cohort, patients with total EULAR/ACR scores ≥10 showed significantly greater contributions from mucocutaneous, immunologic, renal and constitutional domains compared with those scoring <10. (**B**) When the analysis was restricted to ANA-positive patients, immunologic and renal domains emerged as the principal contributors distinguishing patients with scores ≥10 from those below the classification threshold, indicating a stronger dependence on high-weight immunologic and renal involvement for classification attainment

**Table 3:** Clinical and immunologic domain distribution according to EULAR/ACR-2019 classification status.

EULAR/ACR-2019	Fulfilled (*n* = 62, 70.5%)	Not fulfilled (*n* = 26, 29.6%)	*P* value
**ANA positivity**	62 (100)	4 (15.4)	**<0.001**
**Mucocutaneous involvement**	48 (77.4)	13 (50)	**0.011**
**Constitutional manifestations**	33 (53.2)	8 (30.8)	0.054
**Musculoskeletal involvement**	23 (37.1)	10 (38.5)	0.904
**Neuropsychiatric involvement**	13 (21.0)	2 (7.7)	0.131
**Serositis**	10 (16.1)	2 (7.7)	0.293
**Hematologic involvement**	30 (48.4)	10 (38.5)	0.394
**Renal involvement**	25 (40.3)	6 (23.1)	0.122
**Immunologic involvement**	57 (91.9)	15 (57.7)	**<0.001**
**Anti-dsDNA positivity**	33 (53.2)	0 (0)	**<0.001**
**Anti-Sm positivity**	23 (37)	0 (0)	**<0.001**
**Antiphospholipid antibody positivity**	15 (24.2)	1 (3.8)	**0.032**
**Low complement**	43 (69.4)	13 (50)	0.085

Data are presented as *n* (%). Comparisons between groups were performed using χ^2^ or Fisher’s exact test as appropriate. ANA positivity constitutes the mandatory entry criterion for EULAR/ACR-2019 classification. Bolded *P* values <0.05 were considered statistically significant. EULAR: European Alliance of Associations for Rheumatology; ACR: American College of Rheumatology; ANA: antinuclear antibody; anti-dsDNA: anti–double-stranded DNA antibody; anti-Sm: anti-Smith antibody.

Among the 15 unclassified patients, 14 (93.3%) were ANA-negative. Despite a lower mean total score compared with ANA-positive patients (15.6 ± 4.9 *vs* 22.3 ± 7.9), many exceeded the ≥10-point threshold but remained unclassifiable due to the mandatory ANA entry criterion. Major weighted domains were largely absent in this subgroup, while immunologic involvement predominantly driven by hypocomplementaemia represented the predominant domain despite otherwise limited systemic disease.

### Genetic pathway-based differences in the performance of lupus classification criteria

Fulfilment of all three classification criteria differed significantly across genetic pathway subgroups (all *P < *0.05). For EULAR/ACR-2019, classification performance varied markedly by pathway (χ²=13.48, *P = *0.004), being highest in complement pathway defects (89.3%, 25/28) and immune regulation defects (83.3%, 15/18), intermediate in type I interferon pathway variants (66.7%, 4/6) and lowest in nucleic acid metabolism defects (50.0%, 18/36). A similar pattern was observed for SLICC-2012 (χ²=10.22, *P = *0.017) and ACR-1997 (χ²=12.34, *P = *0.006), with complement and immune regulation defects consistently showing the highest fulfilment rates across all three criteria, while nucleic acid metabolism defects demonstrated the lowest performance. Triple-criteria fulfilment was most frequent in complement (78.6%) and immune regulation defects (77.8%) and substantially lower in nucleic acid metabolism disorders (47.2%) and type I interferon pathway defects (33.3%; χ²=10.64, *P = *0.014). Failure to meet any criteria was most common in nucleic acid metabolism disorders (36.1%) and absent in type I interferon pathway variants (χ²=15.76, *P = *0.001). Within each genetic pathway, no significant differences in fulfilment performance were observed across the three classification criteria sets (Cochran’s Q, all *P > *0.05) (Fig. 3).

**Figure 3:**
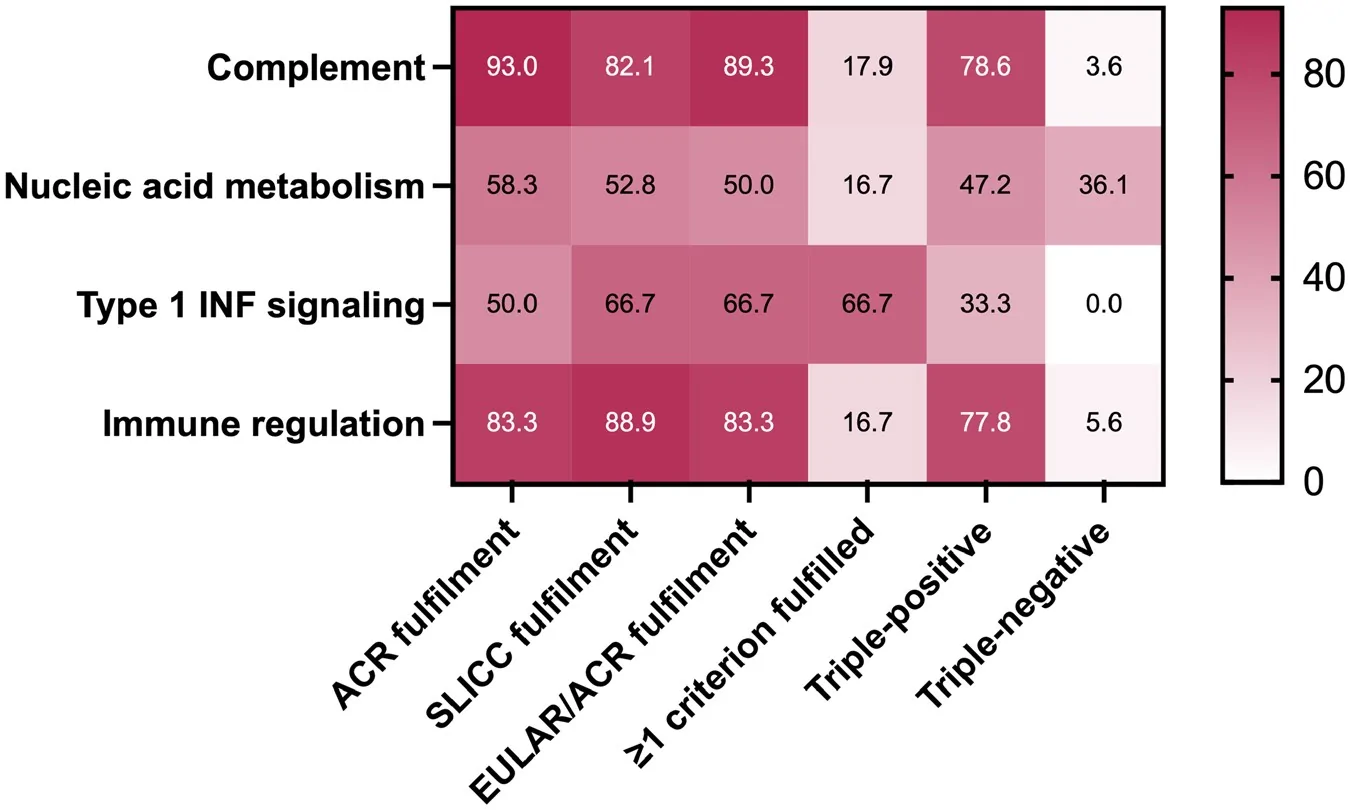
Limitations of current SLE classification criteria across genetic pathways. Heatmap showing fulfilment performance of ACR-1997, SLICC-2012 and EULAR/ACR-2019 classification criteria across genetic pathway subgroups. Percentages represent the proportion of patients within each pathway meeting the specified classification criteria. Colour intensity reflects higher fulfilment performances. Patients with complement pathway defects showed the highest classification concordance, whereas nucleic acid metabolism related disease demonstrated the lowest fulfilment performance and the highest proportion of triple-negative cases

Among 15 patients who did not fulfil any SLE classification criteria, 13 (86.7%) had nucleic acid metabolism and clearance defects. Mucocutaneous, musculoskeletal and hematologic manifestations were accompanied by atypical features, including urticarial and vasculitic/vasculopathic skin lesions, intracranial calcifications/contractures and recurrent infections. Nine patients had low C3 and/or C4, whereas lupus autoantibodies were largely absent. Median diagnostic delay was 24 months (IQR 9–72), compared with 12 months (IQR 3–33.5) in the overall cohort. Despite remaining unclassified, lupus-like manifestations warranted immunomodulatory treatment, including hydroxychloroquine (10/15), glucocorticoids (8/15) and mycophenolate mofetil (6/15); five received JAK inhibitors, all with partial or complete responses. Among 14 patients with longitudinal data, seven had chronic inactive, five relapsing-remitting and two chronic active disease. Median SLEDAI decreased from 4 to 2, median PedSDI was 0 and no deaths occurred.

### Differential domain capture by SLE classification criteria across genetic pathway subgroups

Domain-level analysis revealed consistent pathway-dependent heterogeneity across all three classification criteria. Under ACR-1997, mucocutaneous features particularly discoid rash (*P = *0.015), photosensitivity (*P = *0.004) and oral ulcers (*P = *0.001) were most frequent in complement pathway defects, while haematologic involvement was enriched in interferon-signalling and immune-regulation defects (*P = *0.001). ANA positivity was highest in complement and immune-regulation groups (*P = *0.001). Under SLICC-2012, acute/subacute and chronic cutaneous lupus and oral/nasal ulcers were again most frequent in complement defects (all *P < *0.05), whereas thrombocytopenia predominated in immune-regulation defects (*P = *0.011). Anti-Sm positivity was highest in complement defects (*P < *0.001), anti-dsDNA and complement consumption in immune-regulation and nucleic acid metabolism defects (*P* ≤ 0.005) and direct Coombs positivity in immune-regulation defects (*P = *0.004). Under EULAR/ACR-2019, the same mucocutaneous pattern persisted, with renal domain fulfilment reaching 100% in complement and immune-regulation defects. Anti-dsDNA positivity and complement consumption were predominantly driven by immune-regulation and nucleic acid metabolism defects (both *P < *0.001) (Fig. 4A–C).

**Figure 4:**
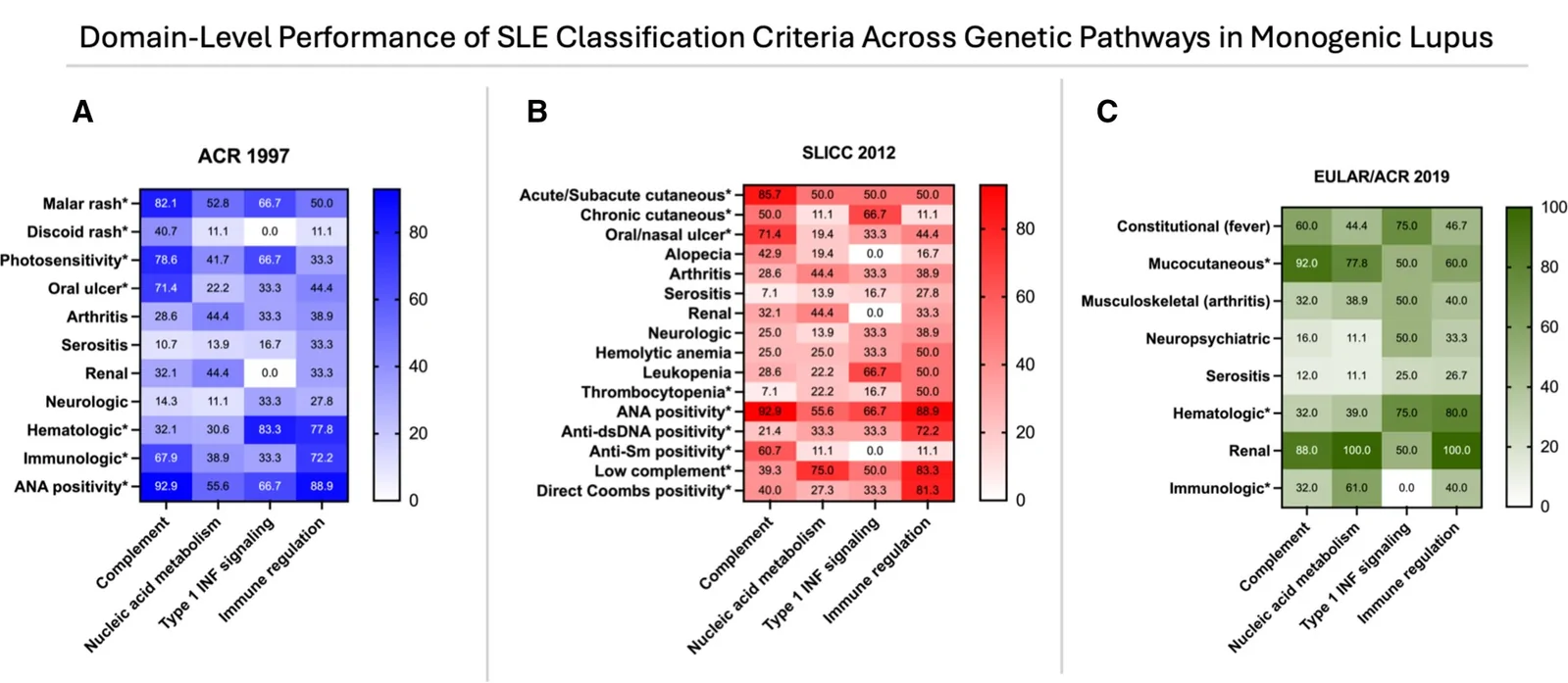
Domain-level distribution of SLE classification criteria across genetic pathways in monogenic lupus. Heatmaps showing the proportion (%) of patients fulfilling classification items and domains across genetic pathway groups. **(A)** ACR-1997: Mucocutaneous and immunologic features were most prominent in complement pathway defects, while hematologic involvement was more frequent in interferon-related and immune dysregulation pathways. **(B)** SLICC-2012: Complement defects showed prominent mucocutaneous involvement and anti-Sm positivity, whereas immune dysregulation and nucleic acid metabolism defects demonstrated stronger immunologic and complement abnormalities. **(C)** EULAR/ACR-2019: Complement defects were associated mainly with mucocutaneous and renal domains, while immune dysregulation and nucleic acid metabolism defects showed greater hematologic and immunologic involvement. Colour intensity represents percentages within each genetic pathway. *P < 0.05 across pathways

EULAR/ACR-2019 analysis was restricted to ANA-positive patients (*n* = 66), of whom 62 (93.9%) met the ≥10-point threshold. The four non-fulfilling patients carried DNASE1L3 (*n* = 2), ACP5 (*n* = 1) and C1R (*n* = 1) variants, presenting with isolated hypocomplementaemia, haematologic–cutaneous or isolated cutaneous involvement, respectively; two fulfilled ACR-1997, one met SLICC-2012, and one met no criteria. Among ANA-negative patients with scores ≥10 (*n* = 15), the nucleic acid metabolism pathway predominated (DNASE1L3 *n* = 8, TREX1 *n* = 2, SAMHD1 *n* = 1), followed by immune-regulation (ACP5, *n* = 2), interferon (IRAK1, *n* = 1) and complement defects (CFB, *n* = 1). Of the 15 patients not fulfilling EULAR/ACR-2019, four met SLICC-2012, five met ACR-1997, two satisfied both and eight met no classification criteria.

## Discussion

This multicentre study, representing the largest nationwide genetically confirmed monogenic lupus cohort, demonstrates that current SLE classification criteria sets perform comparably without clear superiority of any single criterion set. Although, classification performance was primarily driven by genetic pathway rather than by the criteria themselves. Complement and immune-regulation defects were consistently captured, whereas patients with nucleic acid metabolism defects was more frequently missed across all classification criteria sets. Notably, even the most recent EULAR/ACR-2019 criteria failed to classify a subset of genetically confirmed patients despite multisystem involvement, largely due to insufficient weighting of renal and immunologic domains.

Across the cohort, fulfilment of conventional lupus classification criteria was primarily driven by classical mucocutaneous and immunologic domains, with renal involvement and ANA positivity serving as key contributors across all classification criteria sets. ACR-1997 and SLICC-2012 classification criteria sets were largely shaped by mucocutaneous manifestations together with renal and immunologic features, while EULAR/ACR-2019 classification depended predominantly on high-weight immunologic domains in addition to ANA positivity as the entry criterion. Patients lacking these classical domains—particularly those without ANA positivity or major immunologic and renal involvement—were more likely to remain unclassified despite confirmed genetic disease. These findings underscore the central role of mucocutaneous and immunologic features in current classification criteria sets and highlight their limited ability to capture atypical or restricted phenotypes of genetically defined monogenic lupus. Consistent with our findings, Al-Mayouf *et al.* reported that 44 of 49 patients with monogenic lupus (89.8%) fulfilled the EULAR/ACR-2019 classification criteria, yielding a sensitivity of 89.9%. The mean classification score (22.3 ± 8.9) was comparable to that observed in our cohort. In their series, the immunologic domain was the most frequently represented (93.9%), followed by musculoskeletal and renal involvement (57.1% each) and mucocutaneous manifestations (55.1%) Furthermore, 46 of 49 patients (93.9%) fulfilled the SLICC-2012 criteria. Within the SLICC-2012 framework, the immunologic domain was present in all patients (100%), while hematologic, musculoskeletal and renal domains were each observed in 57.1% of cases [9, 16]. These findings are largely consistent with our results and further support the applicability of both EULAR/ACR-2019 and SLICC-2012 criteria in monogenic lupus, with comparable classification performance and domain distribution across independent cohorts.

In this study, classification performance in monogenic lupus was driven predominantly by underlying genetic pathways rather than by differences between existing classification criteria sets. Complement and immune-regulation defects were consistently well captured by all criteria sets, whereas nucleic acid metabolism related disease was frequently under-classified and accounted for most patients who failed to meet any criteria despite confirmed genetic diagnosis. Notably, no classification criteria sets demonstrated clear superiority within individual genetic pathways, indicating that current frameworks share similar pathway-dependent limitations. Domain-level analyses further revealed distinct phenotypic clustering across pathways, with complement defects showing a prominent mucocutaneous and anti-Sm–dominant profile and immune-regulation or nucleic acid metabolism related disease enriched for hematologic involvement, anti-dsDNA positivity, and complement consumption. Together, these findings underscore that existing lupus classification criteria sets incompletely capture the molecular and phenotypic heterogeneity of monogenic lupus and highlight the need for genetically informed, pathway-adapted classification approaches. In line with previous reports, complement pathway defects tend to exhibit a more classical lupus phenotype, whereas other genetic subgroups—particularly immune-regulation and nucleic acid metabolism related disorders—often present with more atypical clinical features [17, 18]. These groups may differ from the conventional clinical manifestations incorporated in current lupus classification criteria, particularly in mucocutaneous and immunologic domains, and may therefore follow a clinical course that diverges from the classical lupus phenotype [3–6, 19–22].

An important question is whether genetically defined patients who do not fulfil conventional SLE classification criteria, particularly those with nucleic acid metabolism and clearance defects lacking immunologic domains, should uniformly be considered to have SLE. Although classification criteria are not diagnostic criteria, the absence of characteristic immunologic features in some patients suggests that ‘monogenic lupus’ may encompass a broader phenotypic spectrum [23]. Importantly, remaining unclassified did not imply clinically insignificant disease: these patients had lupus-like manifestations warranting SLE therapies, with reduced disease activity and limited long-term damage. Treatment was guided primarily by clinical phenotype and organ involvement rather than criteria fulfilment. The longer diagnostic delay in unclassified patients (24 *vs* 12 months overall) may reflect the complexity of these atypical presentations, although this descriptive comparison does not establish causality. While complement and immune-regulation defects more closely recapitulated classical lupus, some nucleic acid metabolism and clearance defects may represent genetically defined immune dysregulation disorders with lupus-like manifestations [3, 24, 25]. Longitudinal genotype–phenotype studies are needed to clarify whether these conditions belong within the lupus spectrum or represent distinct molecular entities.

Our findings reveal two distinct mechanisms underlying misclassification in monogenic lupus under current criteria. First, a small subset of ANA-positive patients with genetically confirmed disease failed to reach the EULAR/ACR-2019 ≥ 10 threshold due to insufficient accumulation of high-weighted domains, often presenting with limited or organ-restricted phenotypes such as isolated hypocomplementemia or cutaneous hematologic involvement. Second, a larger group of patients, predominantly those with nucleic acid metabolism defects, exceeded the ≥10-point threshold yet remained unclassifiable because of ANA negativity, despite substantial immunologic activity. Notably, many of these high-scoring ANA-negative patients were also not captured by SLICC-2012 or ACR-1997, leaving a considerable proportion of genetically confirmed cases unclassified across all classification criteria sets. Together, these observations indicate that current classification criteria sets may fail to recognize both low-domain burden ANA-positive patients and immunologically active but ANA-negative monogenic lupus, emphasizing the need for classification approaches that better integrate genetic background and pathway-specific phenotypes. Similar misclassification patterns have been reported by Al-Mayouf *et al.* in which five monogenic lupus patients failed to meet EULAR/ACR-2019 criteria despite confirmed genetic disease. In their series, three patients with *DNASE1L3* variants were not classified: two due to ANA negativity despite scores above the ≥10 threshold and one due to insufficient cumulative score, while two additional ANA-positive patients with *C1q* deficiency and an *IL-2RB* variant failed to reach the threshold. Notably, four of these five patients were also not captured by the SLICC-2012 criteria, supporting our observation that both ANA negativity and limited high-weighted domain involvement contribute to under-recognition of monogenic lupus across existing classification criteria sets [9].

This study represents the largest genetically confirmed nationwide monogenic lupus cohort to date, enabling detailed genotype–phenotype and pathway-based evaluation of current lupus classification criteria sets within a genetically defined population. The multicentre design and combined item-, domain- and pathway-level analyses provided a robust framework to assess how ACR-1997, SLICC-2012 and EULAR/ACR-2019 criteria perform across molecular subgroups. However, the retrospective nature of the study and heterogeneity in data capture across centres may have led to incomplete documentation of some clinical features. In addition, the absence of a non-monogenic comparator cohort precluded assessment of specificity, limiting interpretation to case-based capture performance, and relatively small numbers in certain genetic subgroups—particularly interferon-pathway defects—may have reduced statistical power for subgroup analyses. Furthermore, the single-country setting may limit the generalizability of our findings, as the distribution of monogenic lupus-associated variants and molecular pathways may differ across populations due to ancestry, consanguinity patterns and founder effects. Validation in larger, ethnically and geographically diverse cohorts is therefore needed to determine the broader applicability of these pathway-specific findings.

## Conclusion

In this large nationwide genetically defined monogenic lupus cohort, current SLE classification criteria sets showed substantial agreement without clear superiority, yet their overall performance remained inadequate, with each individual criteria set failing to classify approximately one-third of genetically confirmed patients. Accordingly, the development of genetics-informed and pathway-adapted classification approaches represents a critical and unmet need in the field. These findings suggest that conventional criteria may not fully represent the phenotypic spectrum of monogenic lupus. Future pathway-informed and genetics-integrated classification approaches may improve recognition, stratification of monogenic lupus in both clinical practice and research settings.

## Data Availability

The data underlying this article are not publicly available due to patient confidentiality and ethical restrictions. De-identified data may be made available from the corresponding author upon reasonable request, subject to institutional and ethical approval.
